# Sir Arthur Downes on Poor-Law Progress

**Published:** 1914-03-21

**Authors:** 


### SIR ARTHUR DOWNES ON POOR-LAW PROGRESS.

### New Theatre at Tynemouth Union.

The Guardians of the Tynemouth Poor-Law Union on Monday last put the finishing touch to their scheme for training, when the operating theatre in connection with their institution hospital was formally opened by Sir Arthur H. Downes, M.D., L.G.B. senior medical inspector for Poor-Law purposes. Erected at a cost of about £650 by Mr. James Carruthers, Tynemouth, from designs prepared by Mr. W. Stockdale, A.R.I.B.A. The scheme comprises three rooms and an entrance lobby; the operating theatre is 17 feet by 15 feet; the anæsthetic room 15 feet 6 inches by 7 feet; and the sterilising room 13 feet 6 inches by 7 feet. All these rooms are approached by polished hardwood doors, with coinage metal fittings.

All floors are executed in polished marble terrazzo, with covered skirtings at the walls, thus presenting a jointless surface. White opalite tiling, relieved with cream bands, has been employed at the wall surfaces, and the angles of the rooms and window openings have all been hollowed and rounded. In connection with the windows special sections of steel casements have been erected. The ventilation system has had special attention; fresh air is introduced to the building after having passed through medicated wool and warmed by radiators, and the vitiated air is drawn out at the ceiling level by a controlled air-pump extractor. In the heating installation all the pipes have been kept out of the rooms, and sufficient heating surface is provided by steam-heating radiators arranged to swing out for easy cleaning.

The Chairman of the Board, Councillor John Frater, in extending a welcome to Sir Arthur Downes, referred to the Royal Commission Report on the Poor Laws. That Commission alluded to the great improvement that had taken place since the issue of the Nursing Order of 1897, which put a stop to pauper patients being employed as attendants in the sick and lying-in wards of workhouses. This great reform in Poor-Law infirmaries was owing to the Memorandum issued on January 29, 1895, by Sir Arthur Downes. Mr. Frater also referred to Sir Arthur's attitude as one of the Royal Commissioners in dissenting from certain findings of the Commission, and stated that had it not been for the issue of his Memorandum then they, as a Board of Guardians, might not have had the privilege of being present there that day.

Sir Arthur Downes expressed thanks for the warm reception accorded to him.

"This admirably designed and well-formed operating theatre, which I now declare open," he proceeded, "is dedicated by the Guardians of Tynemouth to the healing of the sick poor of this great Union. Structurally it is but a small portion of the provision already made, but in significance it may be regarded as the coping-stone of that provision. First, it reminds us of that marvellous advance of surgical knowledge and skill initiated by the discoveries of Pasteur, applied by the practical genius of our own Lister. Secondly, this is a development which is bound up with another great advance. I mean the nursing of the sick. It also provides a landmark of a broadened basis of public assistance. To myself, who have had exceptional opportunities of learning the facts and watching this evolution, it is a continual regret to note how seldom Guardians receive the credit which should be theirs. The legislator is certainly not entitled to assume any merit in these great advances in public assistance to the sick. It is almost incredible, but it is none the less a fact, that, with the exception of London, this country is devoid of any statutory provision for Poor-Law medical relief. I can bear witness, and I do so in common fairness, that nearly all the main improvements in the administration of the English Poor Law have been practically initiated by progressive Boards of Guardians, advised and aided by able officers. If the Central Authority and the Legislature had realised the true import of public feeling, and warnings came very early in the history of the Poor Law of 1834, we should have had long since in this country a broad basis of public assistance, and we should probably have heard little of the specious and misleading clamour to abolish the Poor Law. The main problem of the Poor Law now lies in dealing with the children and in providing for the aged and sick or infirm, and with such a staff and such an equipment Tynemouth will be amply repaid by the good results that must ensue."

